# Fourier transform infra-red spectroscopy and flow cytometric assessment of the antibacterial mechanism of action of aqueous extract of garlic (*Allium sativum*) against selected probiotic *Bifidobacterium* strains

**DOI:** 10.1186/1472-6882-14-289

**Published:** 2014-08-06

**Authors:** Jemma Booyens, Mapitsi Silvester Thantsha

**Affiliations:** Department of Microbiology and Plant Pathology, University of Pretoria, New Agricultural Sciences Building Room 9–10, Lunnon road, Pretoria, 0002 South Africa

**Keywords:** Fourier transform infrared spectroscopy, Flow cytometry, *Bifidobacterium*, Garlic (*Allium sativum*), Probiotic

## Abstract

**Background:**

It is generally reported that garlic (*Allium sativum*) harms pathogenic but not beneficial bacteria. Although numerous studies supporting the alleged garlic effects on pathogens are available, there are limited studies to prove this claim for beneficial bacteria. We have recently shown that garlic exhibits antibacterial activity against probiotic bifidobacteria. The aim of the current study was to elucidate the mechanism of action of garlic clove extract (GCE) on *Bifidobacterium bifidum* LMG 11041, *B. longum* LMG 13197 and *B. lactis* Bb12 using Fourier transform infrared (FT-IR) spectroscopy and flow cytometry.

**Methods:**

Cultures (1 × 10^8^ CFU ml^-1^) were individually incubated for 6 h at 37°C in garlic clove extract containing allicin at a corresponding predetermined minimum bactericidal concentration for each strain. For FTIR, an aliquot of each culture was deposited on CaF_2_ slide and vacuum dried. The slides were immediately viewed using a Bruker Vertex 70 V FT-IR spectrometer equipped with a Hyperion microscope and data analyzed using OPUS software (version 6, Bruker). Spectra were smoothed with a Savitsky-Goly function algorithim, base-line corrected and normalized. Samples for flow cytometry were stained using the Live/Dead *Bac*Light bacterial viability kit L7012. Data compensation and analysis was performed using a BD FACSAria and FlowJo (version 7.6.1).

**Results:**

Fourier transform infrared spectroscopy showed changes in spectral features of lipids and fatty acids in cell membranes, proteins, polysaccharides and nucleic acids. Spectral data as per principle component analysis (PCA) revealed segregation of control and GCE-treated cells for all the tested bifidobacteria. Flow cytometry not only showed increase in numbers of membrane damaged and possibly lysed cells after GCE treatment, but also displayed diffuse light scatter patterns for GCE treated cells, which is evidence for changes to the size, granularity and molecular content of the cells.

**Conclusion:**

Garlic has multiple target sites in bifidobacteria, penetrating the cell membrane and entering the cytoplasm, where it causes changes to carbohydrates, fatty acids, proteins and nucleic acids. These changes, for example, modification of membrane properties, may prevent exposed bifidobacteria from colonizing the intestinal mucosa. Loss of colonization potential would render them less efficient as probiotics.

## Background

Bifidobacteria, used as probiotics, are added to a variety of different food products and pharmaceutical preparations due to their numerous health benefits [1, 2]. In order to provide these health benefits, these bacteria need to be present in sufficient viable amounts within products, as well as be able to attach to and colonize the intestinal mucosa. It is therefore important to test susceptibility of these probiotic bacteria to compounds that may render them ineffective or decrease their capability to perform. Knowledge of the foods or food ingredients that may compromise viability of these bacteria is critical to their success in enhancing consumers’ health. Garlic (*Allium sativum)* is a strong antibacterial agent and it is known to inhibit various pathogenic microorganisms [3].

We have recently revealed sensitivity of bifidobacteria to antibacterial effects of garlic [4], as well as initiated the process of elucidating its mechanism of action on these bacteria. Electron micrographs showed that garlic induced unusual morphological changes in bifidobacteria [5]. It is however not yet known whether garlic inhibition on probiotic cells is confined to the cell membrane or if there are other targets. We anticipate additional targets as allicin, the main active compound in garlic, has been reported to kill pathogens through total inhibition of RNA synthesis, partial inhibition of DNA and protein synthesis, and alteration of the electrochemical ability and induce apoptosis in cells [6–9]. It is also known to affect microbial lipid biosynthesis, signal transduction, as well as react with thiol-containing proteins [10–12].

Fourier transform infrared (FT-IR) spectroscopy is a fairly new technique used to study the entire molecular composition of microbial cells. It can “fingerprint’ the entire cell and detect even the minimal cellular compositional changes that other methods fail to reveal [13]. This is possible because all functional groups of organic molecules are able to absorb IR light [14]. The infrared spectra of bacterial cells is able to reveal the biochemical composition of their cellular constituents which include the cell wall and membrane (composed of phospholipid bilayer, peptidoglycan and lipopolysaccharides), and the cytoplasm (fatty acids, water, nucleic acids, proteins and polysaccharides) [14, 15]. This technique not only offers a rapid and non-invasive alternative to study changes or injury that takes place in bacterial cells, but it also requires minimal sample preparation [11].

Researchers elsewhere have used FT-IR spectroscopy to study the effect of different stress factors, injury or treatment with some antimicrobial compound on compositional changes of internal molecules of bacteria. It has recently been used to investigate the mode of action of bactericidal compounds and to determine changes in bacterial membrane fluidity and membrane phase behaviour in response to environmental stresses [13]. Zoumpopoulou *et al*. [14] used it to detect internal cellular changes in *Salmonella enterica* serovar typhimurium induced by exposure to antimicrobial compounds. Sub lethal thermal injury in *S. enterica* and *Listeria monocytogenes*, and cold stress injury in *Campylobacter jejuni* and *Pseudomonas aeruginosa* have also been identified using FT-IR spectroscopy [11, 15]. Furthermore, it has been used to study chlorine-injured *P. aeruginosa* and *Escherichia coli* in water, radical induced damage of *Micrococcus luteus* and heat-killed *E. coli* O157:H7 in ground beef [16]. In studies closely related to our current study, it has been used to detect sub lethal damage in foodborne pathogens, such as *C. jejuni*, *E. coli* O157:H7 and *L. monocytogenes*, as a result to garlic exposure [12, 17].

Flow cytometry on the otherhand, is a technique that allows rapid analysis of individual cells, and can also simultaneously assess morphological and cellular functions [18]. It has been used to assess bacterial viability of probiotic products, dairy starter cultures [19] and to measure viability changes of different *Bifidobacterium* spp. [18, 20]. Bunthof *et al*. [21] used flow cytometry to assess survival of different probiotic strains after their exposure to bile salt and acid.

This study therefore aimed to investigate the potential effects induced on macromolecules of bifidobacteria by GCE, using FT-IR and flow cytometry, in order to determine and better understand its mechanism and extent of damage on these bacteria.

## Methods

### Culture and garlic clove extract preparation

*Bifidobacterium bifidum* LMG 11041 and *B. longum* LMG 13197 strains (BCCM/LMG culture collection, Belgium) were revived as per manufacturer’s specifications. *Bifidobacterium lactis* Bb12 was obtained from CHR-Hansen, Denmark. All cultures were grown in MRS-cys-HCl broth and incubated at 37°C for 48 h in anaerobic jars containing Anaerocult A gaspacks (Merck Ltd, Modderfontein, SA). The concentration of the cultures was then adjusted to 0.5 McFarland standard (1 × 10^8^ CFU ml^-1^). Both the preparation of garlic clove extract (GCE) and spectrophotometric determination of the concentration of allicin, the major active compound in the extract, were performed as described previously [4]. Garlic clove extract was added to 1 ml of each culture up to a final allicin concentration equivalent to the predetermined minimum bactericidal concentrations of 99.4, 198.7 and 39.8 μg ml^-1^ for *B. bifidum* LMG 11041, *B. lactis* Bb12, and *B. longum* LMG 13197, respectively. Cultures were then incubated at 37°C for 6 h.

### FT-IR spectroscopy

#### Preparation of bacteria

Bacterial cells were recovered from prepared 1 ml broth cultures by centrifugation at 13400 rpm for 15 min. The supernatant was discarded and pellet was washed twice in ¼ strength Ringer’s solution. The pellet was then resuspended in 1 ml distilled water, ready for sample preparation and measurements. The average cell concentration was kept constant at 1 × 10^8^ CFU ml^-1^ in order to generate consistent FT-IR signals. Samples for FT-IR were prepared according to Marcotte *et al*. [22] with slight modifications. A 5 μl aliquot of each bacterial sample was deposited onto a CaF_2_ slide and air-dried at room temperature under low vacuum for 15 min. A further 2 μl of sample was deposited on top of the dried sample to form a homogenous dried film of cells. Samples were analyzed directly after preparation to prevent any changes associated with aging of the samples.

#### Spectral measurements and data analysis

A Bruker Vertex 70 V FT-IR spectrometer (Bruker Optics, Germany), equipped with a Hyperion microscope attachment was used. The bacterial coated CaF_2_ slides were placed under the microscope objective and IR spectra were recorded in transmission mode from 4000 to 850 cm^-1^ at a spectral resolution of 4 cm^-1^. Fifteen spectra for each sample were obtained at room temperature. A total of 45 spectra (3 × 15) for each treatment were obtained. Experiments were performed in triplicate. OPUS software version 6 was used to perform data analysis. Spectra were smoothed with a Savitsky-Goly function algorithim with 25 smoothing points, base-line corrected and normalized. Principle component analysis (PCA) was used on raw data to separate and group control and GCE-treated bacterial spectra to illustrate differences between the two data sets.

### Flow cytometry

#### Preparation of bacteria

Bacterial cells from prepared 1 ml broth cultures were harvested by centrifugation at 13400 rpm for 15 min and the pellet washed twice with ¼ Ringers solution. Cells were resuspended in phosphate buffered saline (PBS) and the concentration adjusted to 10^8^ cells ml^-1^. Live untreated cells in their mid-exponential phase and heat-killed (70°C for 30 min) cells were used as negative and positive controls, respectively.

#### Staining of bacteria

Samples (live, heat killed, and GCE treated) of each *Bifidobacterium* were stained with the Live/Dead BacLight bacterial viability kit L7012 (Molecular Probes, Netherland). Three replicates of each control were individually stained with 1.5 μl (1:20 dilution) of PI, SYTO9 or a 1:1 mixture of SYTO9 and PI. These samples were used to set up the protocol, and to differentiate between live, dead and compromised cells. Flow cytometric detectors and compensation settings for the different quadrants were performed using control samples. Dual staining was also performed for GCE treated samples. All the samples were then incubated in the dark for 15 min. Thereafter, they were centrifuged at 13400 rpm for 10 min and pellets resuspended in PBS and placed on ice before analysis.

#### Data capture and analysis

A BD FACSAria (BD Biosciences, USA), fitted with four argon lasers (488 nm, 633 nm, 405 nm laser and 375 nm near UV laser) was used. BD FACSflow was used as sheath fluid and 10 000 events per sample were counted. Green fluorescence (SYTO9) was detected through a 530 nm, 30 nm-bandwidth band-pass filter which amounts to a range of 675-715 nm (FITC). Red fluorescence (PI) was detected through a 695 nm, 40 nm band-pass filter (Per-C-P), amounting to a range of 515-545 nm. A combination of forward scatter (FSC) and sideward scatter (SSC) was used to discriminate bacteria from background. Experiments were performed in triplicate and data compensation and analysis was performed using FlowJo Version 7.6.1.

## Results and Discussion

### FT-IR spectroscopy

Fourier transform infra-red spectra for most bacteria have four distinguishable regions. Region I (3000-2800 cm^-1^) represents cell membrane fatty acids, with three noticeable peaks (2960, 2925, and 2860 cm^-1^) [11, 14]. Region II (1700-1500 cm^-1^) shows amide I (1650 cm^-1^) and amide II (1550 cm^-1^) bands of proteins and peptides [16]. Region III (1500-1200 cm^-3^) corresponds to fatty acids as well as proteins and phosphate-carrying molecules. Three major peaks at 1455 cm^-1^, 1400 cm^-1^ and 1240 cm^-1^ depict changes to lipids and proteins; carbohydrates and nucleic acids or phospholipids, respectively. Bands at 1080 cm^-1^ are also related to nucleic acids (11). Region IV (1200-900 cm^-3^) shows absorption bands typical of polysaccharides or carbohydrates of microbial cell walls with an absorption peak between 1100-950 cm^-1^ [14, 16].

The spectra obtained for all the tested *Bifidobacterium* strains were similar to those previously described for other bacteria [11, 14, 23]. Strong absorptions were obtained for all four spectral regions (4000-850 cm^-1^) representing the main components of a cell. Control and GCE-treated *Bifidobacterium* samples showed discrepancies in spectra obtained, as discussed in detail for each tested strain below.

#### *Bifidobacterium bifidum*LMG 11041

**Figure 1:**
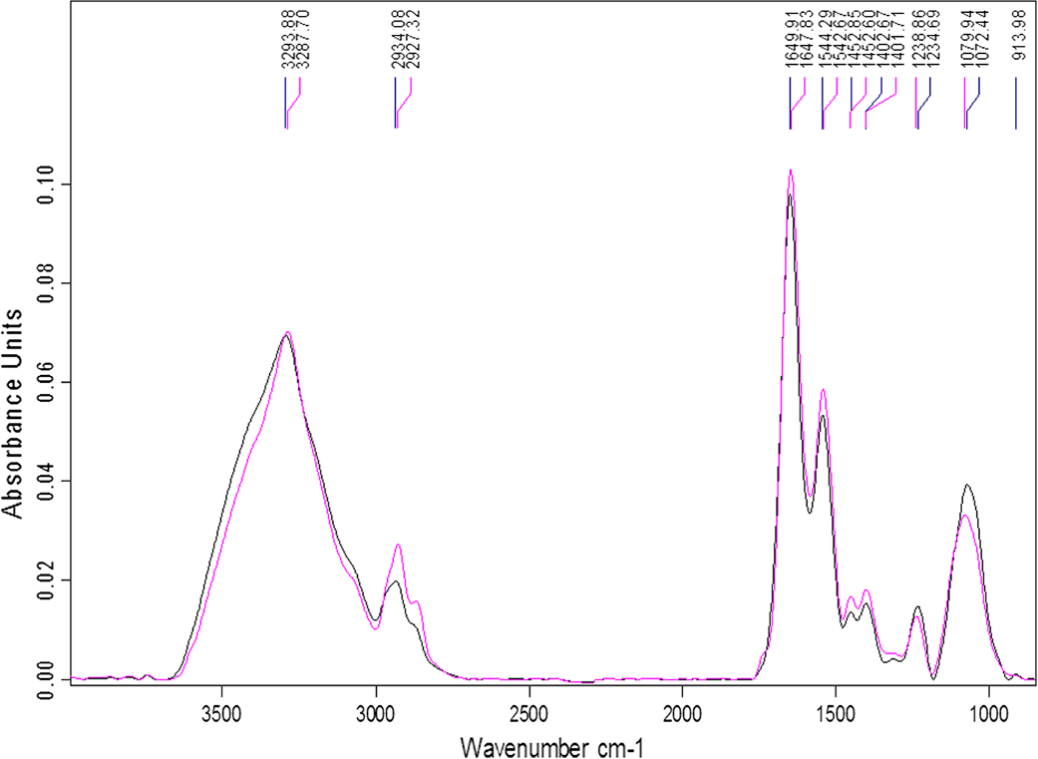
**Fourier transform infra-red spectra of** ***B. bifidum*** **LMG 11041 samples, control: black; garlic clove extract-treated: pink.**

Spectral features of control and GCE treated samples were different (Figure 1). There were shifts at all major peaks, 3285, 2930, 1655, 1550, 1452, 1400, 1238, 1078, and 913 cm^-1^ (Figure 1), indicating changes in the biochemical composition of cells due to exposure to GCE. Shifts at peaks 3285 cm^-1^ indicate changes to proteins and polysaccharides while shifts at peak 2930 cm^-1^ correspond to changes to lipids. In addition, there was an increase in spectral frequency at peak 2934 cm^-1^, which may indicate an increase in membrane fluidity as well as a conformation disorder of the acyl chains of the cell membrane phospholipids [13]. These results suggest a change in properties of cell membranes of treated cells. Minor shifts at peaks 1649 cm^-1^ and 1544 cm^-1^ (Amide I and II) were also observed, with an associated increase in intensity of spectral features of these two peaks for GCE-treated cells compared to the control. This could indicate an increase in polysaccharides, which serve as a mechanism of survival for cells to down regulate functions to save energy and protect themselves from stress [11]. The biggest shifts occurred at peaks 1238 cm^-1^ and 1079 cm^-1^, which represent phosphodiesters and nucleic acids, and carbohydrate regions in the cell wall, respectively. There were major decreases in spectral intensities of GCE treated cells for these peaks, which could indicate a reduction in viable counts, prevention of cell growth or cell death [11]. Allicin, the main active compound of garlic, can readily pass through cell membranes and affect lipid and fatty acid biosynthesis causing changes in viability of cells [10]. Changes in the nucleic acid region corresponds to published literature reports stating that allicin completely inhibits RNA synthesis and partially inhibits protein and DNA synthesis [6]. Furthermore, the spectra for control showed a clear peak at 913 cm^-1^, which disappeared after treatment of cells with GCE. This may indicate damage to the phospholipids in the cell wall. Similar observations were reported for *Escherichia coli* and *Listeria monocytogenes* due to their exposure to garlic [17].

### *Bifidobacterium longum*LMG 13197

**Figure 2:**
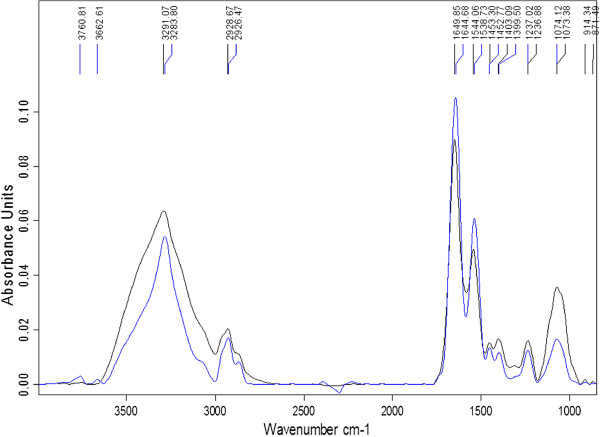
**Fourier transform infra-red spectra of** ***B. longum*** **LMG 13197 samples, control: black; garlic clove extract-treated: blue.**

Similar to what was observed for *B. bifidum* LMG 11041, there were considerable shifts in the spectra at peaks 3291 cm^-1^ and 2928 cm^-1^ for GCE-treated cells (Figure 2). Compared to control samples, prominent changes were observed in region 3600-2800 cm^-1^, with decreased band area and intensities at peaks 3291 cm^-1^ and 2928 cm^-1^ (representing lipids), which could be related to a reduction in cell viability. A decrease in spectral band intensity is an indication of cell death. Similar results were previously reported for *E. coli* O157:H7 that were subjected to cold stress and low nutrient media [11]. Environmental stresses have been shown to induce changes in the cell membrane lipid composition [24]. Furthermore, allicin disrupts microbial lipid and fatty acid formation, thereby causing changes in viability of cells [10]. An increase in intensities of both amide bands for GCE-treated cells was also observed. These cells also showed a decrease in spectral intensities in regions 1490 cm^-1^ – 1260 cm^-1^ and 1074 cm^-1^, coupled with a reduction in band area at peak 1074 cm^-1^, changes usually associated with cell death or cessation of growth [11]. Shifts at peaks 1236 cm^-1^ and 1074 cm^-1^ suggest denaturation of the phosphodiester backbone of nucleic acids, thus damage to DNA and RNA. The peaks observed at 914 cm^-1^ and 871 cm^-1^ for controls were absent in GCE-treated cells, indicating changes to the structure of the bacterial envelope polysaccharides [23].

### *Bifidobacterium lactis*Bb12

**Figure 3:**
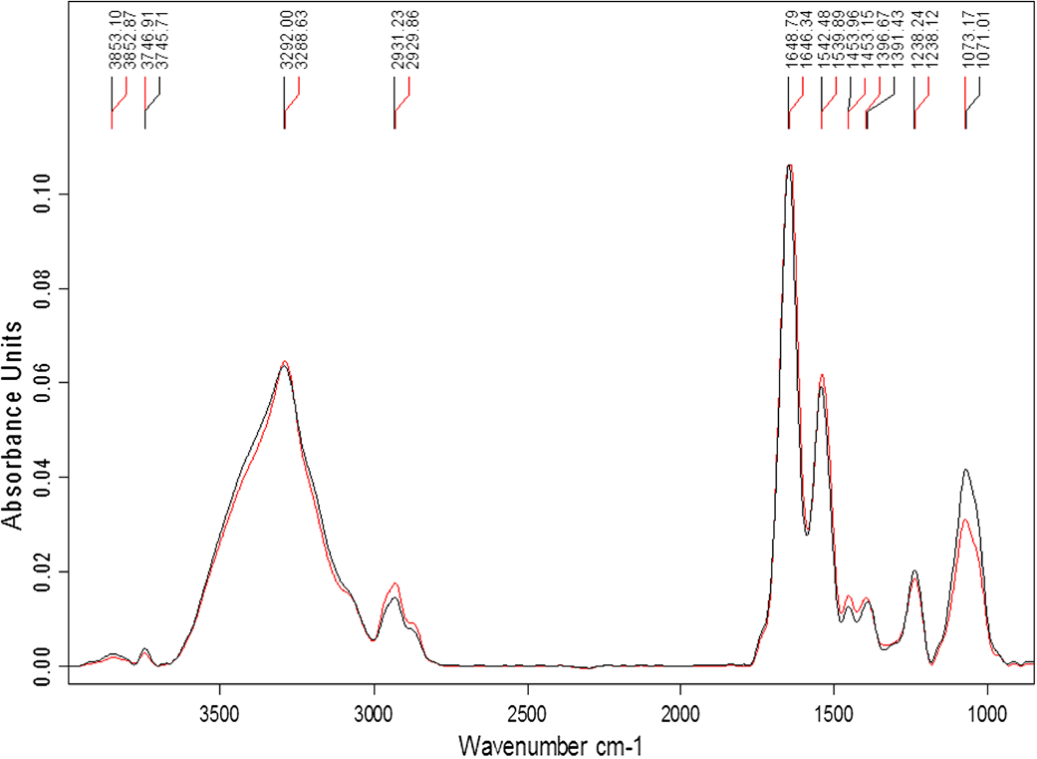
**Fourier transform infra-red spectra of** ***B*** **.** ***lactis*** **Bb12 samples, control (black); garlic clove extract-treated (red).**

There were minimal discernible differences between spectral features for control and GCE treated *B. lactis* Bb12 samples at peaks 2930 cm^-1^ (lipids), 1648 cm^-1^ (Amide I), 1542 cm^-1^ (Amide II), 1453 cm^-1^ (proteins) and 1235 cm^-1^ (phosphodiesters) (Figure 3). The observed visual similarities between FTIR spectra for control and GCE treated *B. lactis* is attributed to its intrinsic resistance of detrimental factors, specifically its relative resilience to antibacterial effects of garlic, compared to other bifidobacteria [4]. However, a slight decrease in spectral intensity around the lipid regions (2929 cm^-1^) and a reduction in intensity around the phosphodiester region (1235 cm ^-1^) for GCE-treated cells compared to controls were noticeable. As already mentioned, these are changes attributed to a decrease in cell viability [11]. According to Al-Qadiri *et al*. [15], a change in the spectral regions at peak 1235 cm ^-1^ indicates nucleic acid denaturation. A major decrease in intensity for this strain was observed at peak 1073 cm^-1^, which is within a region corresponding to nucleic acids. Therefore, these observations once again indicated that GCE altered RNA and DNA, as was observed with the other *Bifidobacterium* strains tested in this study, although the change was less pronounced for this strain, and reported for other bacteria elsewhere, a damage that may eventually lead to cessation of growth or microbial death.

### Principle component analysis of FTIR spectra

Principle component analysis has been extensively used for interpretation of infrared spectra in microbiology, medicine, agricultural and food sciences. It reduces a multidimensional data set to its most dominant features, removes random variation while maintaining the relevant variations between data points [16, 25]. It shows whether there are definite clusters in the data and describes similarities or differences from multivariate data sets [25]. In this study, PCA was performed concurrently for all four FTIR spectral regions described, as well as for the separate regions. Groupings of the spectra representing differences or similarities among the regions were then used to compare molecular compositions of control and GCE-treated bifidobacteria.

**Figure 4:**
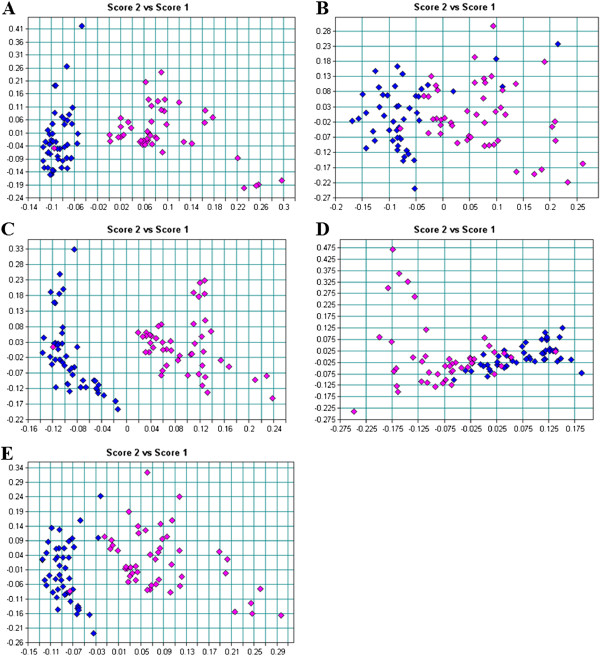
**PCA of the first derivative and multiplicative scatter correction (MSC) of** ***B. bifidum*** **LMG 11041 for A: region I (3000-2400 cm** ^**-1**^ **), B: region II (1800-1500 cm** ^**-1**^ **), C: region III (1500-1200 cm** ^**-1**^ **), D: region IV (1200-900 cm** ^**-1**^ **) and E: all spectral regions (4000-850 cm** ^**-1**^ **), control: blue; garlic clove extract-treated: pink.**

Figure 4 shows the PCA of the first derivative and multiplicative scatter correction of *B. bifidum* LMG 11041. Comparison of whole spectra patterns of the control and GCE-treated samples showed segregation between these two groups of samples (Figure 4E). However, upon closer inspection, distinct separation between clusters in region I (Figure 4A) and III (Figure 4C) were observed than in other regions, with differences better distinguished in region III than region I. There was no distinct clustering for in spectral regions II and IV (Figure 4B and D). These findings show that the most significant changes induced by GCE occurred in the region representing cell structure proteins and phosphodiesters associated with phospholipid bilayers, while other cellular constituents were less affected. This further explains our previous reports of unusual morphological changes for this strain [4].

**Figure 5:**
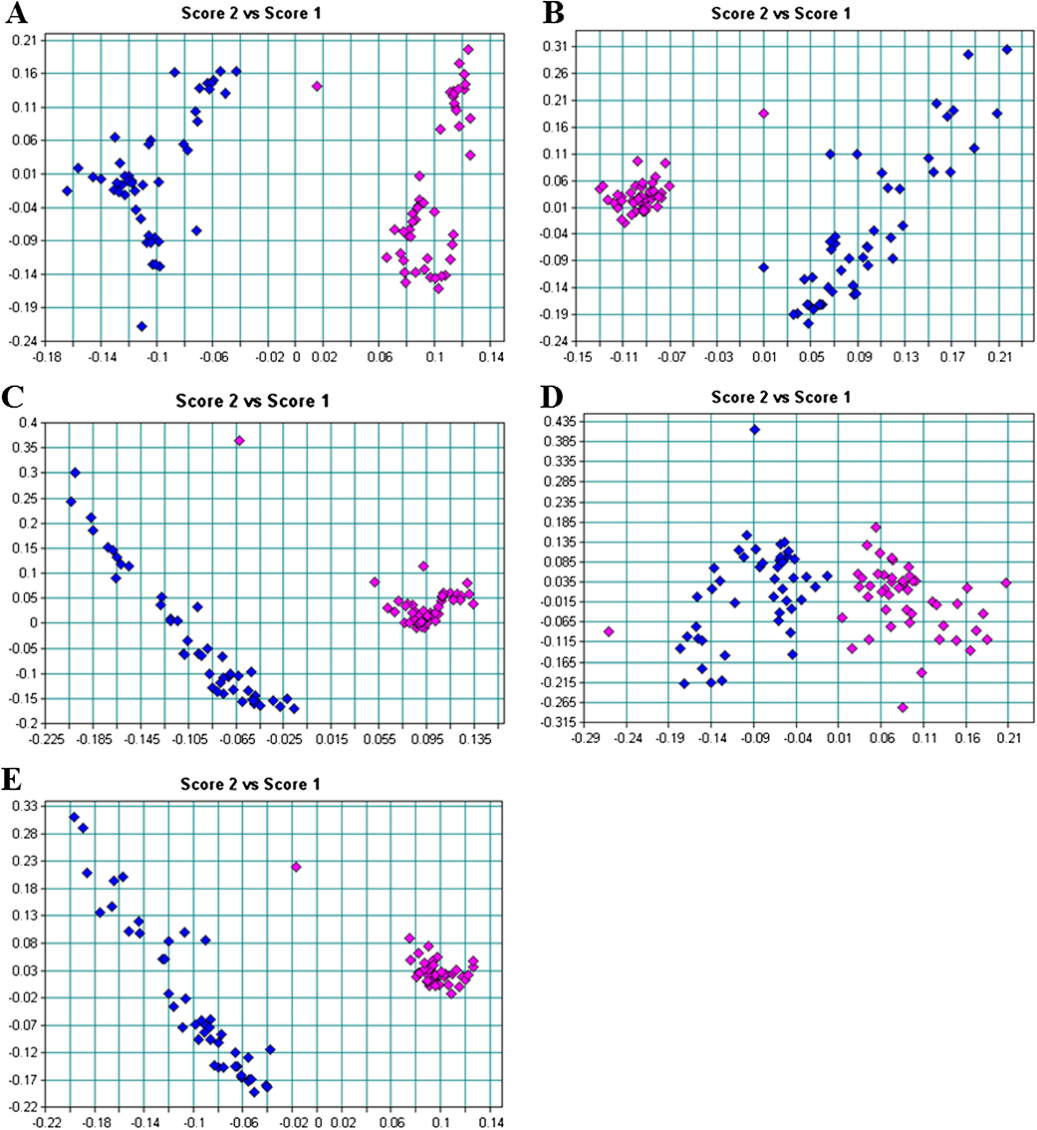
**PCA of the first derivative and multiplicative scatter correction (MSC) of** ***B. longum*** **LMG 13197 for A: region I (3000-2400 cm** ^**-1**^ **), B: region II (1800-1500 cm** ^**-1**^ **), C: region III (1500-1200 cm** ^**-1**^ **), D: region IV (1200-900 cm** ^**-1**^ **) and E: all spectral regions (4000-850 cm** ^**-1**^ **), control: blue; garlic clove extract-treated: pink.**

Separation of spectra between *B. longum* LMG 13197 control and GCE-treated samples over all regions were observed (Figure 5). Major spectral differences were observed in regions I, II and III (Figure 5B-D) than in region IV. This indicated that for *B. longum* LMG 13197, polysaccharides of the cell wall were less affected than the other cellular components. Garlic clove extract caused more biochemical changes in this strain compared to *B. bifidum* LMG 11041.

**Figure 6:**
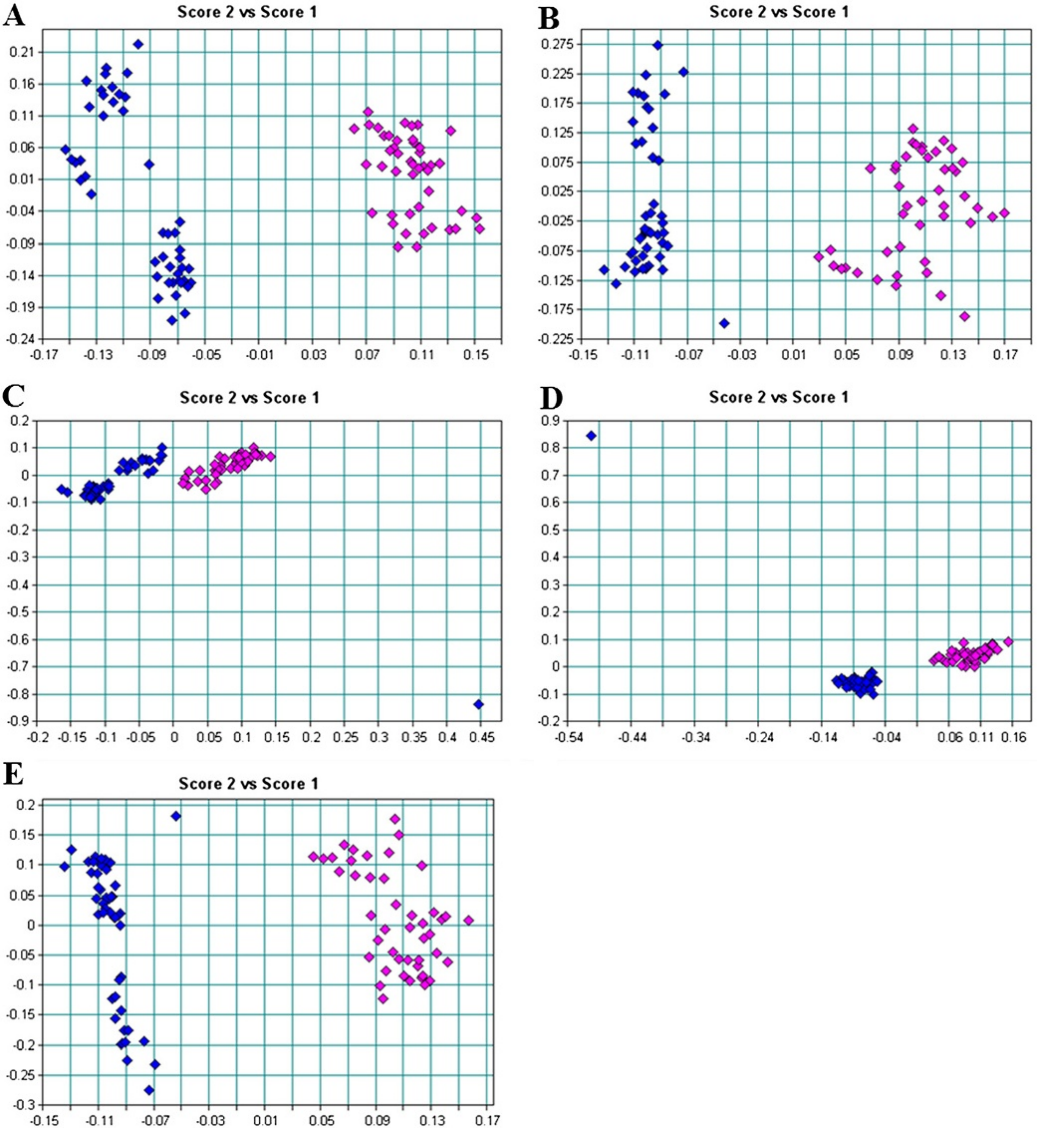
**PCA of the first derivative and multiplicative scatter correction (MSC) of** ***B. lactis*** **Bb12 for A: region I (3000-2400 cm** ^**-1**^ **), B: region II (1800-1500 cm** ^**-1**^ **), C: region III (1500-1200 cm** ^**-1**^ **), D: region IV (1200-900 cm** ^**-1**^ **) and E: all spectral regions (4000-850 cm** ^**-1**^ **), control: blue; garlic clove extract-treated: pink.**

Clear separations with distinct sample clusters were observed for control and GCE-treated *B. lactis* Bb12 throughout all the spectral regions (Figure 6). However, there were fewer differences between control and GCE-treated samples in regions III and IV as these clusters were closer to each other (Figure 6). Significant differences were observed within the lipid and protein regions, indicated by the more isolated clusters (Figure 6B-C). Damage was confined to the cell wall for this strain whereas for the more sensitive strains (*B. bifidum* LMG 11041 and *B. longum* LMG 13197), it extended to the nucleic acids.

In summary, PCA confirmed distinctive features of the FT-IR spectra among *Bifidobacterium* cells, indicated by clear segregations with distinct sample clusters in most if not all of the spectral regions between control and GCE-treated cells. In all the tested cells, exposure to GCE resulted in significant changes in lipids or fatty acids in the cell membrane, structural proteins and phosphodiesters associated with phospholipid bilayer. Noteworthy differences between control and GCE-treated cells were also observed in the amide groups of proteins, the nucleic acids as well as in polysaccharides of the cell walls for *B. longum* LMG 13197 and *B. lactis* Bb12.

### Flow cytometric analysis

**Figure 7:**
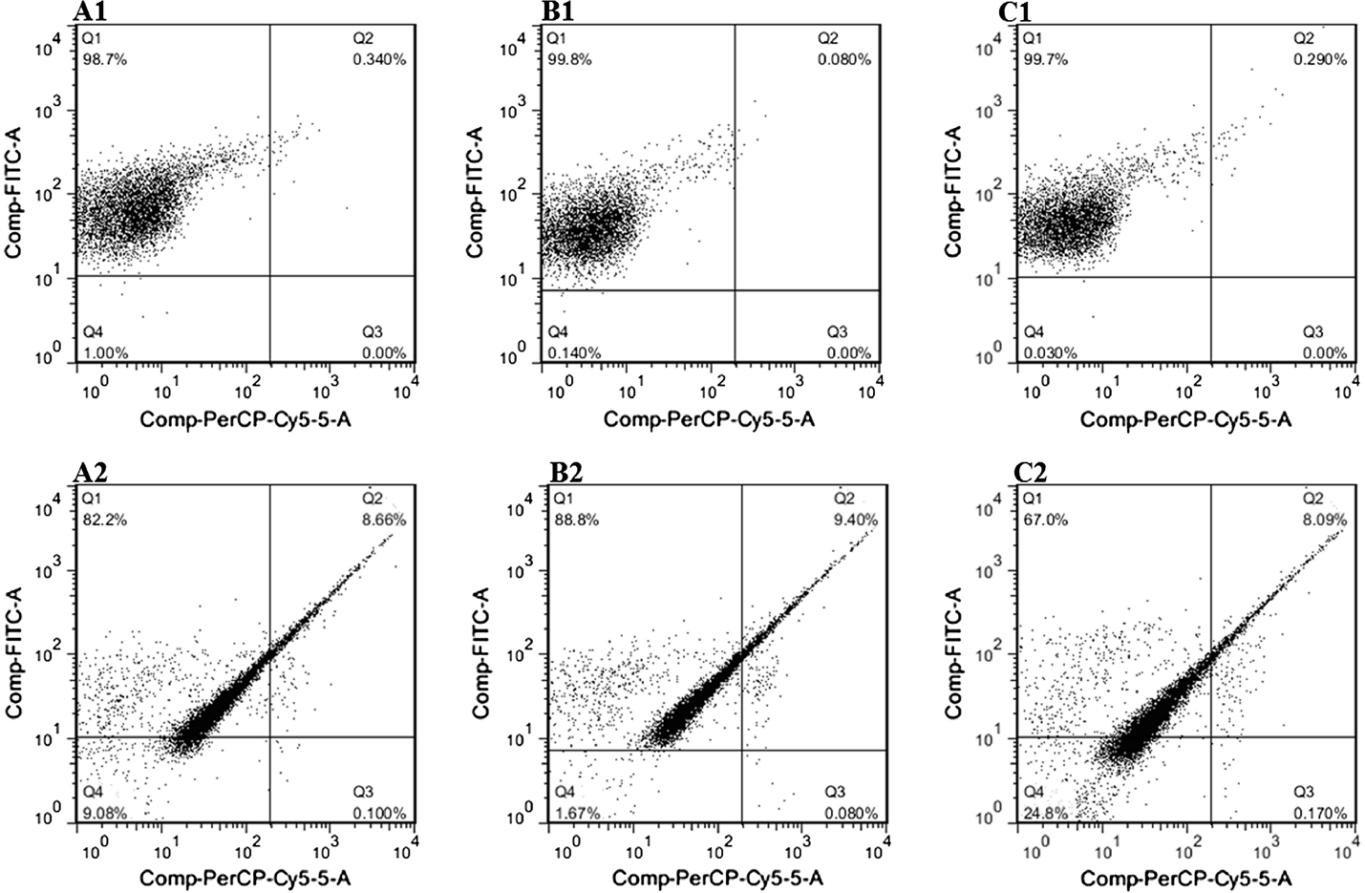
**Flow cytometry dot plots of** ***B*** **.** ***bifidum*** **LMG 11041 (A1) control, (A2) garlic clove extract-treated;** ***B*** **.** ***longum*** **LMG 13197 (B1) control, (B2) garlic clove extract-treated;** ***B*** **.** ***lactis*** **Bb12 (C1) control, (C2) garlic clove extract-treated.**

Double staining of *Bifidobacterium* cells with PI and SYTO9 was used to determine the effect of GCE on the cell membrane integrity, thereby giving an indication of whether the cell is viable, dead or damaged. Intact, undamaged cell membrane excludes the nucleic acid dye, PI, while damage permeabilizes it to this stain, which upon entry into the cells binds to nucleic acids, resulting in red fluorescence. Propidium iodide is therefore used as a marker for dead cells. SYTO9 stains all cells in a population, whether they are alive or dead [26]. It is however displaced by PI from the nucleic acids of damaged cells due to the higher affinity of the latter for DNA [21]. These stains have been used in studies on lactic acid bacteria to differentiate between healthy and damaged cells [19, 21, 27]. Figure 7 depicts the dot plots obtained for the different *Bifidobacterium* strains before and after treatment with GCE. Different quadrants were set to represent the following populations of cells: quadrant 1 (Q1): live SYTO9-stained cells, quadrant 2 (Q2): double-stained membrane damaged cells, quadrant 3 (Q3): dead PI-stained cells and quadrant 4 (Q4): unstained cells. There were noticeable differences between control and GCE treated cells for all the tested *Bifidobacterium* strains. The percentage of viable cells of *B. bifidum* LMG 11041 cells decreased by about 17% after GCE treatment while increases of 8.32, 0.1 and 8.08% were observed in Q2, Q3 and Q4, respectively (Figure 7, A1 and A2). *Bifidobacterium longum* LMG 13197 followed a similar trend to *B. bifidum* LMG 11041. The viable cell population decreased by 11% after GCE treatment, whereas number of damaged cells in Q2 increased by 9.3% (Figure 7, B1 and B2). The major difference between *B. longum* LMG 13197 and *B. bifidum* LMG 11041 was that an increase in the percentage of unstained *B. bifidum* cells, was less than that observed for *B. longum* by 7.4%. These results suggest that this strain was less susceptible to antibacterial effects of GCE. The viable population of *B. lactis* Bb12 was only 67% after GCE treatment compared to 99.7% for control sample. However, for this strain, the reduction in percentage of viable cells was accompanied by a substantial increase in percentage (24.77%) of unstained cells (Figure 7, C1 and C2), an increase higher than that observed for each of the other two strains by more than 15%. This result did not correspond to data obtained by FTIR, which indicated less damage to *B. lactis* Bb12. However, the percentage of cells having membrane damage was comparable to that obtained for the other two strains. Flow cytometric data results confirmed that all three *Bifidobacterium* strains tested were susceptible to GCE. An increase in percentage of cells in Q2 is evident of progressive cell damage and membrane deterioration. Similar flow cytometric data for bifidobacteria exposed to bile salt stress was obtained by Ben Amor *et al*. [28]. Ananta *et al*. [27] also found cell membrane damage using flow cytometry to analyze viability of *Lactobacillus rhamnosus* after spray drying.

A definite reason for the presence of unstained cells in Q4 is unknown, but it could be attributed to one or a combination of the following. Firstly, unstained cells could correspond to the cells that have undergone severe lysis and thus lost their nucleic acids, thereby rendering them unstainable [29]. These ‘ghost cells’ have been described by other researchers elsewhere [30, 31]. Previous studies have also reported that garlic causes cell lysis. Kim *et al*. [32] reported cell wall lysis in *Listeria monocytogenes* cells treated with garlic shoot juice. Allicin, the major active compound of garlic, has been reported have the ability to pass through phospholipid membranes, causing cell membrane damage and eventually cell lysis and death [8]. Secondly, these could be cells that clumped together or formed interlaced chains which, according to Hayouni *et al*. [33], may decrease staining accuracy.

Differences in shape and density of the populations’ light scatter patterns before and after exposure to GCE were also observed for all the tested *Bifidobacterium* strains. After GCE treatment the light scatter patterns became less diffuse and more concentrated, indicating a change in size, granularity and molecular content of the cells (Figure 7). Similar results were also observed in *Candida albicans* by Grannoum 1988, where the change in structure and integrity of the outer membrane was attributed to a decrease in lipid content of the membrane in the presence of garlic [32]. A different light scatter pattern obtained after exposure of cells to GCE could be due to a change in size or cellular structure and external morphology, whereby cells change from rod to coccoid shape. We have recently reported a change of bifidobacteria from rod to cocci shaped cells with cross- walls due to treatment with GCE [4]. Similar results were reported by Schenk *et al*. [34] for *E. coli* and *L. innocua* cells exposed to UV-C light. Young [35] also showed that typical rod-shaped *Bifidobacterium* spp. became coccoid under stress.

## Conclusions

The mechanism of action of garlic towards bifidobacteria is similar to that which was reported for pathogenic bacteria. Bacterial growth inhibition and death occurs due to modifications to secondary structure proteins, fatty acids and phospholipids in the cell membrane as well as nucleic acids. Changes to the membrane properties may therefore prevent these probiotic *Bifidobacterium* strains from colonizing the intestinal mucosa and thereby hinders them from delivering the beneficial effects. Hence, we suggest that precaution be taken with simultaneous use of probiotic bifidobacteria and garlic. Future studies could focus on sorting the GCE modified cells and comparing their physiological properties to that of their original counterparts not exposed to garlic, to establish whether these damaged but live cells can still deliver their probiotic effects to the consumer. Furthermore, the effect of food matrices on allicin levels, as well as how long allicin persists in the gastrointestinal tract, needs to be investigated.
